# Effects of a School-Based Health Intervention Program in Marginalized Communities of Port Elizabeth, South Africa (the KaziBantu Study): Protocol for a Randomized Controlled Trial

**DOI:** 10.2196/14097

**Published:** 2019-07-11

**Authors:** Ivan Müller, Danielle Smith, Larissa Adams, Ann Aerts, Bruce P Damons, Jan Degen, Stefanie Gall, Zaahira Gani, Markus Gerber, Annelie Gresse, Darelle van Greunen, Nandi Joubert, Tracey Marais, Siphesihle Nqweniso, Nicole Probst-Hensch, Rosa du Randt, Harald Seelig, Peter Steinmann, Jürg Utzinger, Christina Wadhwani, Cheryl Walter, Uwe Pühse

**Affiliations:** 1 Department of Sport, Exercise and Health University of Basel Basel Switzerland; 2 Nelson Mandela University Port Elizabeth South Africa; 3 Novartis Foundation Basel Switzerland; 4 Swiss Tropical and Public Health Institute Basel Switzerland; 5 University of Basel Basel Switzerland

**Keywords:** anthropometry, cardiovascular, cognitive function, diabetic complications, children’s health, marginalization, physical activity, physical fitness, schools, South Africa

## Abstract

**Background:**

The burden of poverty-related infectious diseases remains high in low- and middle-income countries, while noncommunicable diseases (NCDs) are rapidly gaining importance. To address this dual disease burden, the *KaziBantu* project aims at improving and promoting health literacy as a means for a healthy and active lifestyle. The project implements a school-based health intervention package consisting of physical education, moving-to-music, and specific health and nutrition education lessons from the *KaziKidz* toolkit. It is complemented by the *KaziHealth* workplace health intervention program for teachers.

**Objectives:**

The aim of the *KaziBantu* project is to assess the effect of a school-based health intervention package on risk factors for NCDs, health behaviors, and psychosocial health in primary school children in disadvantaged communities in Port Elizabeth, South Africa. In addition, we aim to test a workplace health intervention for teachers.

**Methods:**

A randomized controlled trial (RCT) will be conducted in 8 schools. Approximately 1000 grade 4 to grade 6 school children, aged 9 to 13 years, and approximately 60 teachers will be recruited during a baseline survey in early 2019. For school children, the study is designed as a 36-week, cluster RCT (*KaziKidz* intervention), whereas for teachers, a 24-week intervention phase (*KaziHealth* intervention) is planned. The intervention program consists of 3 main components; namely, (1) *KaziKidz* and *KaziHealth* teaching material, (2) workshops, and (3) teacher coaches. After randomization, 4 of the 8 schools will receive the education program, whereas the other schools will serve as the control group. Intervention schools will be further randomized to the different combinations of 2 additional intervention components: teacher workshops and teacher coaching.

**Results:**

This study builds on previous experience and will generate new evidence on health intervention responses to NCD risk factors in school settings as a decision tool for future controlled studies that will enable comparisons among marginalized communities between South African and other African settings.

**Conclusions:**

The *KaziKidz* teaching material is a holistic educational and instructional tool designed for primary school teachers in low-resource settings, which is in line with South Africa’s Curriculum and Assessment Policy Statement. The ready-to-use lessons and assessments within *KaziKidz* should facilitate the use and implementation of the teaching material. Furthermore, the *KaziHealth* interventions should empower teachers to take care of their health through knowledge gains regarding disease risk factors, physical activity, fitness, psychosocial health, and nutrition indicators. Teachers as role models will be able to promote better health behaviors and encourage a healthy and active lifestyle for children at school. We conjecture that improved health and well-being increase teachers’ productivity with trickle-down effects on the children they teach and train.

**Trial Registration:**

International Standard Randomized Controlled Trial Number (ISRCTN): 18485542; http://www.isrctn.com/ISRCTN18485542

**International Registered Report Identifier (IRRID):**

DERR1-10.2196/14097

## Introduction

### Background

Children’s health and well-being are influenced by cultural, environmental, and socioeconomic factors as well as living conditions and social and community networks [1]. In low- and middle-income countries (LMICs), infectious diseases remain an important public health problem [2-4] with negative impacts on child development [5]. Over 200 million children are infected with parasitic worms (helminths) [6,7] leading to chronic infections causing abdominal pain, diarrhea, and anemia, and may impair cognitive and physical development [8], which in turn might result in reduced fitness and work productivity [9]. In addition, helminth infections can negatively impact children’s nutritional status [10].

Although helminth infections and other neglected tropical diseases (NTDs) do not feature prominently in the burden of disease statistics of South Africa, some NTDs are common in disadvantaged populations, especially among children of poor communities [11]. The nutritional status of school children from poor neighborhoods is adversely affected by food outlets in close proximity to the schools. Indeed, many school children routinely purchase unhealthy foods from local vendors and tuck shops that are generally low in nutritional value, often refined, processed, and of low fiber content [12]. In a 12-country study (Australia, Brazil, Canada, Colombia, Finland, India, Kenya, People’s Republic of China, Portugal, South Africa, United Kingdom, and United States of America) [13], South African children showed the highest intake of sugar-sweetened beverages [14]. Schools located in poor communities in South Africa are part of the National School Nutrition Program, where members of the community, usually unemployed parents, are employed as food preparers. They do not have any food- or nutrition-related qualification.

A deprived socioeconomic environment can put children at risk of malnutrition resulting in growth retardation [15]. Studies have shown that malnutrition is associated with stunting and poor cognitive development, resulting in a low intelligence quotient, cognitive delays, and negative impact on motor development [15]. This, in turn, negatively affects children’s ability to concentrate, process information, and focus on academic tasks [16]. Children from low socioeconomic status (SES) families are also less likely to have access to health care or health insurance [17]. Together, this leads to a greater risk of illness, school absence, and ultimately poor academic performance and life prospects [18]. These deficiencies, caused mainly by the socioeconomic environment, can prevent school-age children from realizing their full potential and perpetuate a vicious cycle of poverty and poor health.

In addition, noncommunicable diseases (NCDs) are a rapidly evolving public health problem worldwide, especially in LMICs, imposing a growing burden on population health [2,19] including that of children [20]. Urban African populations have moved toward a disease profile similar to western countries, with increasing proportions of deaths attributed to chronic, lifestyle-related diseases [20]. The coexistence of under- and overnutrition has resulted in a double burden of nutrition-related diseases in Africa [21]. Children may, already at a young age, develop risk factors predisposing them to NCDs in adulthood [22,23]. Hence, children are at risk of compromised health because of a dual burden of disease, which may hamper their development and well-being [2,24]. Potential drivers of this double burden may be related to the shift in dietary habits and reduced energy consumption. This dual burden constitutes a large and growing challenge for health systems in African countries.

With up to 80% of all chronic diseases, stroke, and diabetes being preventable through healthy nutrition and regular exercise, more emphasis should be placed on prevention and awareness campaigns [25]. Physical education (PE) plays a critical role in holistic health education of the child. A randomized controlled trial (RCT) with Swiss elementary school learners (first and fifth graders) has shown that a 1-year school-based intervention can markedly improve physical activity and fitness, while simultaneously reducing obesity [26]. Regular physical activity contributes to the development of physical competence and fitness, as well as to the cognitive, social, and emotional development of the child [27]. As a rule of thumb, children should undertake at least 60 min of moderate-to-vigorous physical activity (MVPA) daily [28].

The Healthy Active Kids South Africa Report Card (2018) has shown that many children, particularly from marginalized communities, do not achieve the minimal daily requirements of MVPA [14]. Schools play an important role in making a meaningful contribution to the goal of achieving the recommended daily physical activity guidelines by incorporating PE lessons, among others, into the school curriculum. One plausible strategy to promote children’s health is through school-based health promotion programs. An attempt by a Swiss-South African research team to increase health literacy in South African children at school was the *Disease, Activity and Schoolchildren’s Health* (DASH) project [4]. The study focused on grade 4 children and the creation of an enabling school environment. The intervention program consisted of 4 main components, including (1) a medical examination and anthelmintic treatment, (2) micronutrient supplementation in the form of a nutrient-dense paste enriched with protein, essential vitamins (vitamin A), minerals, energy, and essential fatty acids, (3) health education (eg, hygiene and healthy nutrition), and (4) physical activity (dancing and playful games).

**Figure 1:**
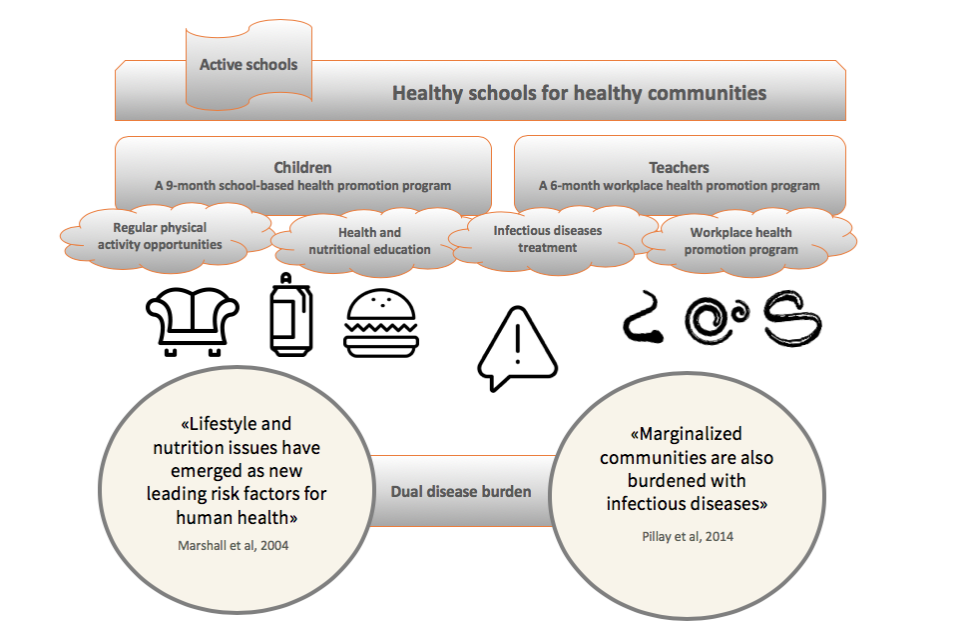
A conceptual framework of the *KaziBantu* study.

Our experiences with the DASH project also revealed that many South African teachers are at risk of cardiovascular diseases [29,30]. This insight was confirmed in a representative sample of South African educators (n=21,307) in public schools. Educators reported high stress levels, and there were significant associations between stress, lack of job satisfaction, and stress-related illnesses [31]. In South Africa, NCDs among adults have steadily increased. Indeed, although 42.90% (256,645/598,240) of deaths in 2005 were attributable to NCDs, the proportion rose to 57.40% (262,096/456,612) in 2016 [32]. Furthermore, in 2017, more than 1.8 million cases of diabetes were recorded in South Africa, representing 5.41% (1,826,100/33,762,000) of the adult population [33]. The project *Healthy Schools for Health Communities* presented here addresses this dual burden of disease, both in school children and teachers in South Africa (Figure 1).

### Rationale

Having identified the potential for health status improvement among teachers and knowing the importance of teachers as role models in the education process of children, teachers will also participate in the proposed research project by involving themselves in a workplace health intervention. We will capitalize on the experiences from the aforementioned DASH project by scaling up the intervention program and monitoring and improving the efficacy and effectiveness of the intervention program. The goal of the *KaziBantu* project is to assess the impact of a school-based health intervention package on communicable diseases, risk factors for NCDs, health behaviors (beliefs and actions relating to health and well-being), and psychosocial health in primary school children in disadvantaged communities in Port Elizabeth, South Africa. In addition, we aim to test a workplace health intervention targeted at teachers.

## Methods

### Ethical Approval and Considerations

Ethical approval for the study has been received from the following ethics committees in Port Elizabeth, South Africa: (1) The Nelson Mandela University Ethics Committee (reference #H18-HEA-HMS-001; obtained on 26 March 2018), (2) Eastern Cape Department of Education (obtained on 9 May 2018), and (3) Eastern Cape Department of Health (reference #EC_201804_007; obtained on 5 June 2018). The study is registered at the ethical review board of the Ethics Committee Northwest and Central Switzerland (EKNZ; reference #R-2018-00047; registered on 1 March 2018).

On the basis of a uniform study information sheet, the investigators will explain to each participant (children and teachers) the purpose of the study, procedures involved, expected duration, and potential risks and benefits. Participation is voluntary, and hence, participants can withdraw at any time without any further obligations. All participants will be provided with an information sheet and a consent form describing the study. Individual medical information obtained during this study will be treated confidentially. Subject confidentiality will be ensured by utilizing subject identification code numbers to correspond to treatment data in password-protected computer files. For data verification purposes, authorized representatives of the EKNZ and the Nelson Mandela University Human Ethics Committee may require direct access to parts of the clinical records relevant to the study, including participants’ medical history.

### Study Area

**Figure 2:**
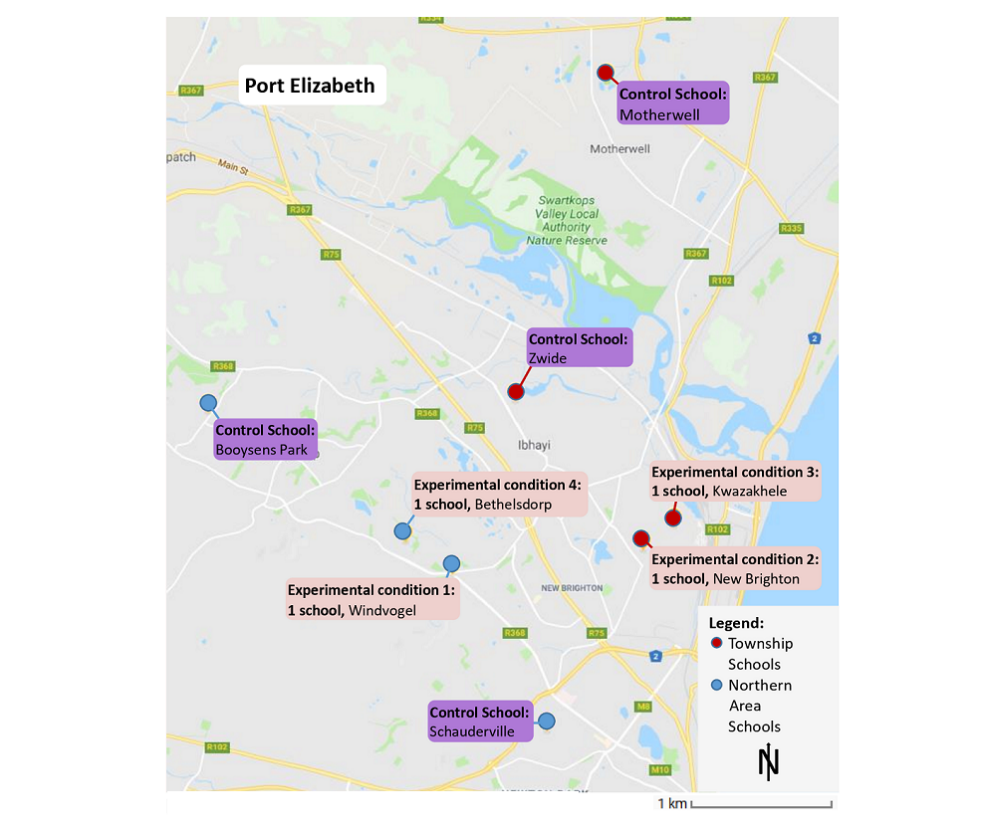
Study area (Port Elizabeth, South Africa) and location of the 8 schools participating in the *KaziBantu* study. Source: Kartendaten, AfriGID (Pty) Ltd.

The study will be conducted in historically black and colored primary schools in Port Elizabeth townships (Motherwell, Zwide, Kwazakhele, and New Brighton) and northern areas (Schauderville, Bethelsdorp, Windvogel, and Booysens Park), which form part of the Nelson Mandela Bay Municipality (Figure 2). These schools and communities are characterized by poverty and high unemployment rates. They represent the typical institutional and teacher-related PE barriers faced by the schools [34], including (1) shortage of qualified, accountable, and engaged PE teachers; (2) PE is marginalized as priority–it lost its standalone subject status in 1997 and is placed within the life skills and life orientation learning area, as more importance is given to other (examinable) subjects; (3) teachers lack the ability to integrate PE with other study areas within the life skills and life orientation subject (personal and social well-being, creative arts, and PE); (4) large class sizes; (5) insufficient and inadequate infrastructure and equipment; and (6) safety and security challenges.

### Study Design

**Figure 3:**
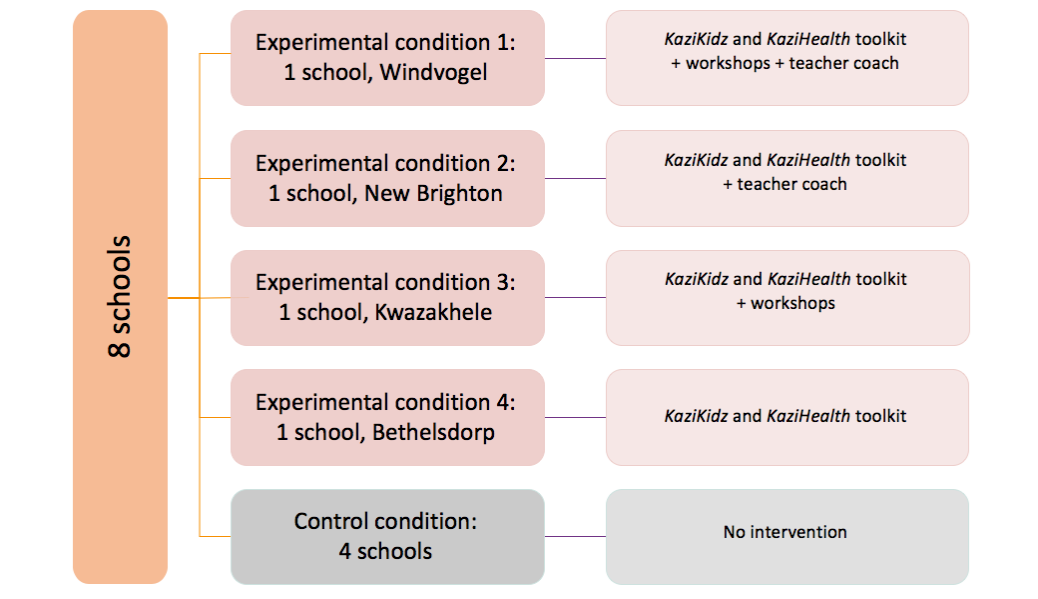
A pictorial display of the *KaziBantu* study design.

The intervention arm targeting school children is designed as a 36-week RCT, including an intervention group (4 schools) and a control group (4 schools). The 4 intervention schools, assigned through randomization, will be further allocated randomly to the following intervention conditions: all schools will receive the teaching materials (*KaziKidz* and *KaziHealth*), but the components workshop and coaching will be assigned as follows: (1) teaching materials only, (2) teaching materials plus workshops, (3) teaching materials plus coaching, and (4) teaching materials plus workshops plus coaching (Figure 3).

The 4 remaining schools will be assigned to the control group. In the control schools, there will be documentation of routine PE and sports in school.

The primary comparison will be made between the 4 intervention schools and the 4 control schools to assess the benefit of teaching materials. Secondary comparisons will be between teaching materials plus coaching and teaching materials without coaching or teaching materials plus workshop and teaching materials without workshop. In view of the factorial design of our study, each comparison group consists of 2 schools.

**Figure 4:**
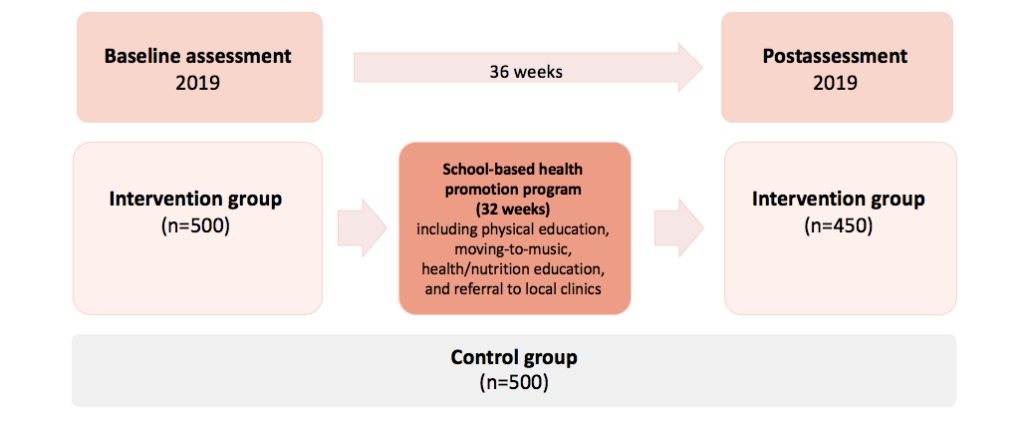
*KaziBantu* study design of testing the *KaziKidz* teaching material.

By focusing on change in quantitative outcomes from baseline to follow-up, preexisting differences between schools should play less of a role. Although the intervention covers grades 1 to 7, in each school, 1 class each from grades 4, 5, and 6 will be randomly selected for evaluation of the intervention. After completion of the baseline assessment, children of the intervention schools will take part in a school-based health promotion program (32 school weeks, 1 PE lesson of 40 min per week, 1 moving-to-music lesson of 40 min per week, 3 health education lessons, and 3 nutrition education lessons of 40 min per year across the whole study period). The follow-up will be after 36 weeks (Figure 4). Qualitative data on the feasibility and acceptability of the intervention measures will also be collected from teachers through focus group discussions (FGDs).

**Figure 5:**
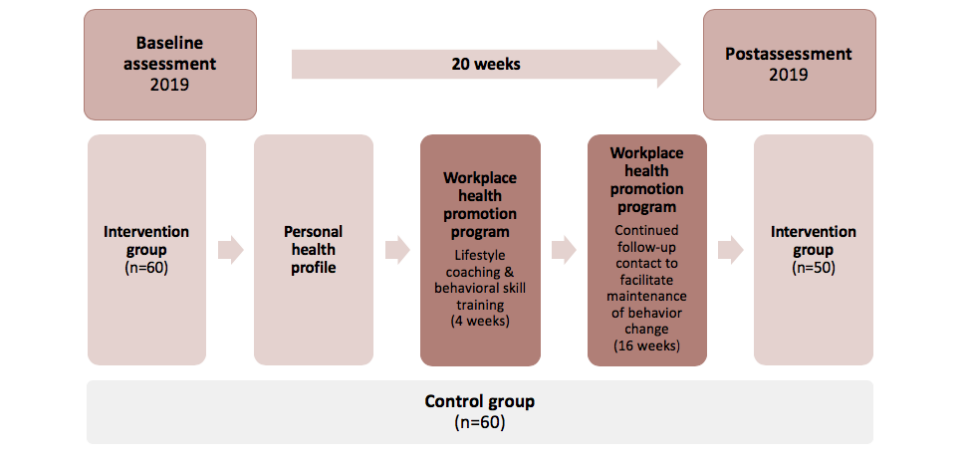
*KaziBantu* study design of testing the *KaziHealth* tools.

For teachers, the study is designed as a 20-week RCT (Figure 5). The baseline assessment will also be offered to the teachers in the control schools. Intervention schools will be randomly assigned to the 4 different combinations of the additional components. After completion of the baseline assessment, all teachers will be informed about their personal health profile, providing an overview of cardiovascular health markers and mental health parameters. For each parameter, established internationally accepted cut-off and normative values will be used to estimate teachers’ health risks.

The intervention program consists of the 3 main components discussed below.

#### Component 1: KaziKidz and KaziHealth Teaching Material

It is a holistic education and instructional tool designed for primary school teachers. This teaching material was pilot tested at 2 elementary schools in the Port Elizabeth area in August 2018. Feedback from teachers was obtained and the material revised accordingly. Through the implementation of 3 content pillars—(1) PE, (2) moving-to-music, and (3) health and hygiene and nutrition education lessons—the toolkit aims to enhance children’s overall health in disadvantaged South African primary schools. The *KaziKidz* teaching material consists of lesson plans within each of the 3 content pillars. The lessons have been designed in line with South Africa’s Curriculum and Assessment Policy Statement. Ready-to-use assessments can be found at the end of each section, which may be integrated into formal assessments of children’s performance and can complement the school's academic curriculum. The purpose is to lead children through content, games, and activities, partly supported by music and conducted in a joyful manner that encourages and promotes a healthy lifestyle throughout childhood and into adolescence. *Kazi* (an animated active mascot, designed to encourage children to participate in *KaziKidz*) and lesson plans will guide teachers through the teaching material. We expect that by using the *KaziKidz* teaching material, teachers will contribute to further the health and well-being of the children they teach and educate.

- Physical activity: Regular physical activity opportunities (1 PE lesson of 40 min per week) will be incorporated into the main school curriculum in grades 1 to 7 over 32 weeks of the school year. A physical activity–friendly school environment will be created. These interventions are designed toward improving children’s physical activity levels and positively affecting their psychosocial well-being.
- The moving-to-music classes have been designed to promote physical activity through song and dance. The music utilized was developed by professional musicians from the Nelson Mandela University and is locally known and age-appropriate. Weekly lessons of 40 min each are designed with easy-to-follow illustrations that allow teachers to instruct without participating physically in the lessons. Schools or teachers who have a sound system available can make use of movement songs that have been created with cues specifically tailored to the lessons. Options for creating music through drums or any other form of percussion or clapping hands are also provided. Within the lessons, direct speech is used to address the children for easy application [35].
- Health, hygiene, and nutrition education: A series of classroom-based lessons have been developed [36]. School children will be educated on the prevention and treatment of intestinal parasite infections, such as proper hygiene, sanitation habits, and the importance of consuming clean water and food. By addressing these factors and educating children about appropriate health and hygiene behaviors, both the teachers and the school children are at a reduced risk of infection. Another series of classroom-based lessons will help to increase awareness about the importance of healthy nutrition. The South African National School Nutrition Program attempts to address micronutrient deficiencies and alleviate short-term hunger by providing food that supplies at least one third of the daily energy requirements of a child. To complement this, the nutrition education lessons (3x 40-min lessons per grade for grades 1-7) should bring dietetics closer to the learners in a playful way and encourage sustainable healthy eating habits throughout the learners’ lives. In addition, an analysis of the schools’ feeding program will be done to identify ways to improve their present diet. The food preparers in schools will also be trained in basic nutrition and hygiene during preparation of the school meals as unhygienic circumstances and poorly prepared meals can lead to infections and low nutrient intake [37].

**Figure 6:**
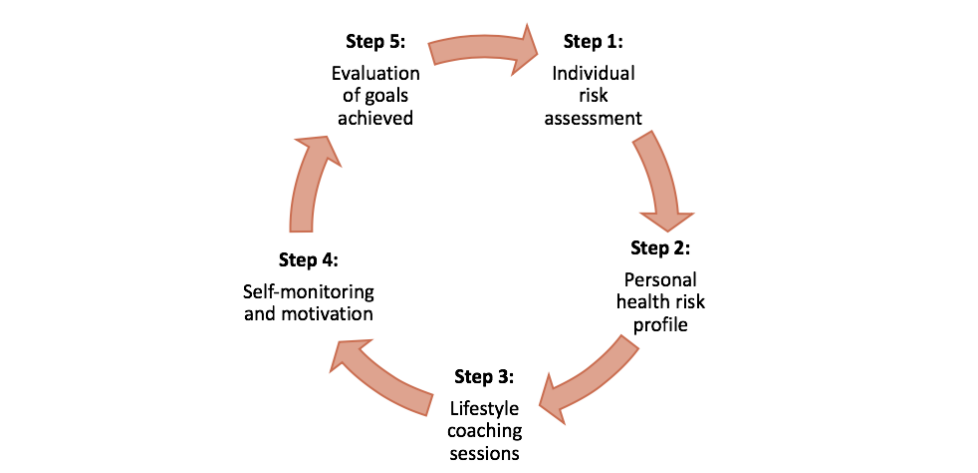
The 20-week workplace health promotion program for teachers.

*KaziHealth* is a workplace health promotion program that aims to educate and improve health behaviors among teachers. The program starts with an individualized health risk assessment followed by face-to-face lifestyle coaching sessions and self-monitoring and motivation through the *KaziHealth* mobile app. All teachers willing to participate in the program will undergo a comprehensive health risk assessment. In addition, teachers of the intervention schools will have the option to participate in a 20-week workplace health promotion program (Figure 6).

#### Component 2: Workshops

Teachers of 2 schools will participate in workshops for both *KaziKidz* and *KaziHealth.* The teaching content (lessons and assessments) of *KaziKidz* will be explained to the teachers before the implementation of the teaching material (2 sessions of 90 min each, as well as practical demonstrations and instruction at schools) and for *KaziHealth*, individually tailored lifestyle coaching workshops (2 sessions of 90 min each). The workshops will be relatively small (maximum of 20 teachers per workshop) and led by health professionals specializing in physical activity promotion, diet, nutrition, and psychosocial health. Furthermore, education, motivation, and self-monitoring will be provided through the *KaziHealth* mobile app [35] to assist individuals in making healthier lifestyle choices and decrease health risks. The *KaziHealth* mobile app [35] integrates 3 lifestyle interventions; namely, physical activity, nutrition, and stress management to guide individuals in achieving personal health goals. To test the efficacy of the workplace health promotion program over time, teachers will be assessed a second time after 20 weeks.

#### Component 3: Teacher Coaches

In the 2 schools where teachers will be offered coaching, trained sports students from the Department of Human Movement Science at the Nelson Mandela University will act as teacher coaches assisting the teachers in teaching and ensuring that the intervention is implemented in the schools correctly and as intensively as planned. Furthermore, they will also monitor the intervention process.

### Sample Size and Randomization

Assuming that the prevalence of obesity varies across schools according to a log-normal distribution with a mean value of 3% and a SD of 2%, 125 children per school in each of the 8 schools would provide a prevalence estimate between 1.5% and 5% with a probability of 95%. Under this assumption, 95% of school-specific prevalence would range between 0.8% and 8.2%. Hence, our aim is to recruit 125 children per school.

The power calculation for the intervention study is based on the change in a quantitative outcome variable from baseline to follow-up. We denote the SD of its change across schools and children by sigma (σ). Assuming an intervention effect size of 0.5 x σ and an intraclass correlation of .04 for the clustering of individual changes within schools (corresponding to a random effect SD of 0.2 x σ), 400 children in the 4 intervention schools (ie, 100 children per school) participating in baseline and follow-up, and 400 children in the 4 control schools would provide over 85% power to observe a statistically significant difference in the mean change of the respective outcome variable between intervention and control schools at the 5% level.

Enrollment of schools will be done by the local research team. To prevent contamination of the intervention effects, schools rather than classes were randomized in January 2019. Before randomization, schools were divided into 2 geographic groups; namely, township areas and northern areas, each containing 4 schools. Township areas are predominantly inhabited by black Africans and northern areas by colored people (after an apartheid-era classification, which refers to people from a multiracial ethnic background and can include persons of Khoi and San origin).

The randomization into intervention and control schools was done separately in each of the 2 groups so that each group was assigned 2 intervention and 2 control schools. To keep the design as balanced as possible, the 4 intervention schemes (ie, teaching materials only, teaching materials plus teacher workshops, teaching materials plus teacher coaching, and teaching materials plus teacher workshops plus teacher coaching) will be assigned in such a way that the intervention schools of 1 group will get teaching materials plus either teacher workshops or teacher coaching. Randomization will allow to determine which of the 2 groups gets which of the 2 pairs of intervention schemes. Sequentially numbered, opaque, sealed envelopes will be used for the assignment of the intervention arms to the schools.

### Study Participants

The effect of the *KaziKidz* teaching material will be evaluated in 1 randomly selected class in grades 4, 5, and 6 (=intermediate phase) in each of the 8 study schools (interventions are randomly assigned to any of the 4 northern area or 4 township schools) even though *KaziKidz* teaching material will be offered to all classes in grades 1 to 7 as part of the life skills and life orientation courses in the school curriculum.

For *KaziHealth*, all teachers from the 8 schools will be invited to participate in the program. All participating teachers will undergo the full health risk assessment, and teachers at the intervention schools will have the option to participate in the 20-week intervention. The teachers from the control schools will be offered the intervention program after the completion of the study.

### School Selection, Participant Recruitment, and Written Informed Consent

South African public schools are classified into 5 groups, with quintile 5 representing the least poor and quintile 1 representing the poorest. The quintiles are determined through the national poverty table developed by the treasury [38]. Areas are being ranked on the basis of income levels, dependency ratios, and literacy rates in the area. The quintile ranking of a school determines the no-fee status of the school and the amount of money that a school receives from the government, with the poorest schools receiving the greatest per-child allocation. Approximately 200 principals and/or representatives from 349 quintile 3 primary schools (no-fee paying schools) of the Nelson Mandela Bay Municipality attended information-sharing sessions at the Eastern Cape Department of Education in October 2018. The intention was to be inclusive and invite as many interested principals as possible to inform them of the study. A total of 64 responses were received from interested schools; however, only 8 of the responses (representative of typical quintile 3 primary schools) matched the following criteria:

1. Geographical location and representation of the target communities: *township areas* inhabited predominantly by black African people and the *northern areas* inhabited by predominantly colored people; both these communities needed to be represented equally.
2. Spoken language (IsiXhosa, Afrikaans, or English).
3. Commitment by school principal to support the project activities.

The school authorities will be informed about the project and asked for their interest and consent. Interested schools will be visited, and the investigators will consult with the school administrators to find out if the school environment is conducive for conducting the study. Principals and teachers from selected schools will be informed about the objectives, procedures, and potential risks and benefits of the study. Teachers, children, and parents or guardians will be informed and teachers and children invited to participate in the study. Before enrollment, a participant information sheet will be provided in English, IsiXhosa, or Afrikaans (local languages) to all potential participants and in case of the children, their parents or guardians. For the evaluation part of the study, oral assent of each participating child will be obtained, whereas written informed consent will be obtained from parents or guardians and teachers. Participation is voluntary; hence, children and teachers can withdraw anytime without any further obligations.

Potential participants will be enrolled in the project for evaluation purposes if they meet the following inclusion criteria: (1) are willing to participate in the study, (2) have a written informed consent (for children by a parent or guardian), (3) are not participating in other clinical trials during the study period, and (4) do not suffer from severe medical conditions, as determined by qualified medical personnel. Approximately 1000 grade 4 to 6 school children, aged 9 to 13 years, and approximately 60 teachers from 8 primary schools will be recruited during the *KaziBantu* baseline survey in early 2019.

### Assessment Methods

**Figure 7:**
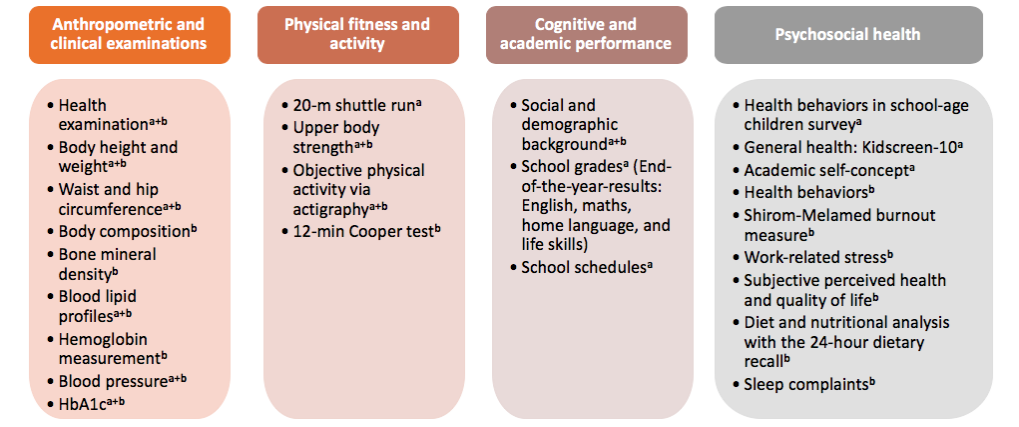
Measurements and tests performed among school children (a) and teachers (b) in the *KaziBantu* study.

Primary outcomes for the *KaziKidz* testing battery include (1) anthropometric and clinical examinations, (2) physical fitness and self-reported and objectively assessed activity, (3) cognitive and academic performance, and (4) questionnaire for assessment of psychosocial health. Primary outcomes for the *KaziHealth* testing battery include (1) anthropometric and body composition assessments, (2) clinical examinations, (3) self-reported and objectively assessed physical activity and physical fitness, and (4) questionnaire results from psychosocial health assessment. Further measures include diet and nutritional analysis with the 24-hour dietary recall. Secondary outcomes for both tests are gender, ethnicity, SES, age, weight, and height. Figure 7 summarizes the assessment methods to be utilized in this study. For baseline and follow-up surveys, the same scientifically recognized procedures will be selected and conducted by professional staff, adhering to standardized, quality-controlled protocols.

### *KaziKidz* Assessment Protocol

#### Anthropometric Measurements

The anthropometric measurements are as follows:

1. For each participant, body weight and height will be measured by standing on a digital weighing scale and against a stadiometer with back erect and shoulders relaxed, recorded to the nearest 0.1 kg and to the nearest 0.1 cm, respectively. Age- and gender-specific height or height-for-age and weight-for-age z-scores will be calculated from the current Centers for Disease Control and Prevention (CDC)/World Health Organization (WHO) growth reference data. Body mass index (BMI) and specific z-scores will be calculated as follows: (1) BMI=weight (kg)/height (m)^2^, (2) BMI for children older than 5 years, an indicator for weight-for-height proportion (WHO growth reference for children older than 60 months) [20], (3) height-for-age, an indicator of growth disorders (WHO growth reference for children older than 60 months), and (4) weight-for-age.
2. A measuring tape will be used to determine the waist circumference of the participant, measured midway between the rib cage and the iliac crest on a gender-appropriate basis. After measuring the hip circumference, the waist-to-hip ratio will be calculated, a risk indicator for heart disease (ie, the smaller the waist in comparison with the hips, the lower the risk of heart disease) [39].

#### Questionnaires

To gather information on children’s social and demographic background, SES, self-perceived stress, school satisfaction, academic self-concept, self-reported physical activity behavior, and general health status, the following questionnaires will be applied:

1. The demographic data and SES of each participant will be determined.
2. The KIDSCREEN-10 will be implemented to determine children’s physical and psychological well-being, moods and emotions, self-awareness, autonomy, parenting and family life, financial resources, peers and social support, school environment, and bullying. The questionnaire comprises 10 points and has proven to be a valid tool for assessing the psychosocial health of children aged 8 to 18 years [40-42].
3. A total of 3 items from the Health Behavior in School-age Children survey [43] will be used to assess individual perceived stress, school satisfaction, and academic self-concept. Learners will be asked how they perceive the pressure, including from homework, related to school [44]. To estimate school satisfaction, children will be asked to respond to the question: *How do you feel about school at present*?
4. Children will also be asked questions about their physical activity behavior, including sports participation, being physically active during school hours, and type of play during school hours and in their free time. Information will be collected over a 7-day period. The questions are adjusted using the Physical Activity Questionnaire for Children, an instrument used to gain insights into general levels of physical activity throughout the elementary school year for children attending grades 4 to 8, aged between 8 and 14 years [45].

#### Clinical Examinations

Clinical examinations will include:

1. The children’s health review will include a detailed history and physical examination. Self-reported health status will focus on intestinal symptoms, including abdominal pain and changes in bowel movements. In addition, we will assess children’s evolution of cognitive and physical development. The physical examination is directed toward evidence of anemia (eg, conjunctival pallor), abdominal conditions (eg, hepatomegaly and splenomegaly), and evidence of pulmonary hypertension (eg, jugular venous pressure and cardiac auscultation).
2. Regarding high blood pressure detection, each participant’s blood pressure will be measured 3 times after the participant has been seated for 5 min with a calibrated Omron digital blood pressure monitor (Omron M6 AC model; Hoofddorp, The Netherlands). The cuff is wrapped around the left arm so that only a finger can fit between the cuff and arm. The bottom of the cuff is placed about 4 cm above the elbow with the palm facing up, while the blood pressure is taken. For children, a cuff size of 17 to 22 cm will be used (Omron CS2 Small Cuff; Hoofddorp, The Netherlands). As the first measurement often results in higher values, the average of the second and third measurements will be utilized to estimate systolic and diastolic blood pressure. To analyze the data, children will be categorized into a normotensive, prehypertensive, or hypertensive group, based on percentiles, taking into account the age, sex, and height of the children (normotensive: less than the ninetieth percentile; prehypertensive: at or above the ninetieth percentile to at or below the ninety-fifth percentile; and hypertensive: at or above the ninety-fifth percentile).
3. For determination of the full blood lipid profile (total cholesterol, low-density lipoprotein cholesterol [LDL-C], high-density lipoprotein cholesterol [HDL-C], triglycerides, non-HDL cholesterol [non-HDL], cholesterol high-density lipoprotein ratio [C-HDL ratio]), glycated hemoglobin (HbA1c) affecting diabetes, and a point-of-care (POC) instrument (Alere Afinion AS 100 Analyzer, Abbott Technologies; Abbott Park, United States of America) will be used, providing results within 8 min. The HbA1c level reflects the average plasma glucose concentration levels over the last 8 to 12 weeks. After the participant’s fingertip is cleaned with an alcohol swab, a nurse will prick the fingertip with a safety lancet and gently squeeze out 2 drops of blood. The first drop will be wiped away, and the second drop will be collected for analysis. Before the assessments, all machines will be tested and calibrated with controls.

#### Physical Fitness Tests

For the purpose of this study, selected tests from the Eurofit fitness battery [46] will be utilized:

1. Cardiorespiratory fitness of children will be measured with the 20 m shuttle run test by Léger et al [47]. In brief, a 20 m flat course, measured by tape and marked with cones will serve for the test. A total of 10 tracks are set. The prerecorded sound signals are played to the children, and they are prompted for the test run in 2 intervals (2x 20 m). Once the children are familiar with the test procedures, they are invited to run back and forth in groups of 10, following the preset pace of the sound signals. Starting at a speed of 8.5 km/h, the frequency of the signal is gradually increased so that the speed increases by 0.5 km/h from 1 min to the next. If children cannot follow the signal and do not reach the 20 m line for 2 consecutive intervals, they will be asked to stop the test and the distance traveled (in full laps) will be recorded. To calculate cardiorespiratory fitness, the number of laps is converted to a speed value, and along with the participant’s age, used in the formula provided by Léger et al [47] to estimate the maximal oxygen uptake (VO_2_ max; ml x kg^-1^ x min^-1^).
2. Upper body strength will be determined using the handgrip resistance test, which measures the maximum isometric grip force. The field investigator will demonstrate how to grip the dynamometer. Each participant will have 1 preliminary trial per hand (with a 30-sec rest in between) to grip the dynamometer as hard as possible. In addition, the dominant hand will be noted. The participants will remain in a standard bipedal pose with their shoulder adducted and neutrally rotated, elbow flexed at 90 degrees, forearm in neutral position holding the Saehan hydraulic dynamometer (MSD Europe BVBA; Tisselt, Belgium) without making contact with any body part. The dynamometer will be adapted to the hand size of each participant, and the maximum readings of 6 trials (measured to the nearest 0.1 kg, 3 trials per hand) will be recorded. The highest score will be used as the final result. Higher values indicate better performance.

#### Objective Activity Measurements

Physical activity behavior will be assessed with an ActiGraph wGT3X-BT accelerometer [48]. Participants will be instructed to wear the device at all times (except during activities involving water contact) for 7 days around the hip. The measured period will include 5 school days and 2 weekend days. Devices will run on the most recent firmware version (version 1.9.2 at the time of writing) and will be initialized with the ActiLife version 6.13.3 (Actigraph LLC) at a sampling rate of 30 Hz. Analyses will be performed using the ActiLife software.

#### Cognitive Performance

In cooperation with the schools, the school exam grades for the following subjects will be obtained: English, mathematics, home language, and life skills. The sum score of these 4 subjects will be used to estimate the academic achievements. In addition, we will obtain school schedules to monitor the overall academic progress of the children. A school schedule is a quarterly summative tool used by schools to measure and track the progress of the learners, across all their subjects, in an academic year. In addition to tracking the child’s progress in the grade, the school schedule is used at the end of the academic year to determine whether the child will proceed to the next grade or be retained in the present grade.

#### KaziHealth Assessment Protocol

At baseline and follow-up testing, a comprehensive health risk assessment by health care personnel will be performed on the participating teachers via the *KaziChat*, a comprehensive health assessment tool, which will be used to capture and interpret all assessed health parameters. Internationally accepted cut-off and normative values will be used to rate each tested parameter based on a traffic light model. A personal health risk profile will be generated, with easy-to-understand explanations of the tested parameters as well as further referrals to a general practitioner, if needed.

### Anthropometry and Body Composition

Utilizing the same protocol as for *KaziKidz*, each participant’s body weight and height will be measured to calculate the BMI. Utilizing the same protocol as for *KaziKidz*, waist and hip circumferences will be measured to determine waist-to-hip ratio, a risk indicator for heart disease [39]. Bone mineral density and body fat percentage will be measured with the Discovery Hologic Dual-Energy X-ray Absorptiometry (DXA) QDR 4500A (APEX System Software Version 4.0.2) by a qualified radiographer. Pregnant individuals, individuals who underwent investigations using radioisotopes in the previous 10 days, and individuals with internal metal artifacts will be excluded from the DXA scan. Calibration will be conducted before testing, using the quality check test. Participant’s height, weight, gender, birth date, and ethnicity will be entered before the participant is instructed to lay supine on an open X-ray table within specified position boundaries. The participant will be instructed to lay still and breathe normally while the scan is being conducted, a process that takes approximately 7.5 min.

#### Clinical Measures

The clinical measures are as follows:

1. A detailed family and medical history will be taken from each participant by a health care professional. Current and previous signs or symptoms of cardiac disease (eg, myocardial infarction, palpitations, and arrhythmias), NCDs (eg, hypertension, dyslipidemia, and diabetes) and psychological conditions (eg, headaches, sleep disorders, and depression) will be recorded. The Physical Activity Readiness Questionnaire will be used to determine whether medical clearance from a general practitioner will be required before the physical fitness assessment [49].
2. Each participant’s blood pressure will be measured 3 times after the participant has been seated for 5 min with a calibrated Omron digital blood pressure monitor (Omron M6 AC model; Hoofddorp, The Netherlands) for the detection of prehypertension and hypertension. A medium or large adult cuff size, 22 to 32 cm or 32 to 42 cm, respectively (Omron Medium and Large Cuff; Hoofddorp, The Netherlands) will be used depending on the participant’s arm circumference. The same protocol as indicated for *KaziKidz* will be followed to determine the final systolic and diastolic blood pressure values.
3. Dyslipidemia and glycosylated hemoglobin will be tested with a POC instrument (Alere Afinion AS 100 Analyzer, Abbott Technologies; Abbott Park, United States of America) using a full lipid profile (TC, LDL-C, HDL-C, TG, non-HDL, and C-HDL ratio) and HbA1c test, respectively. The same protocol used for *KaziKidz* in this regard will also be applied for assessing these variables.
4. For the detection of anemia, the hemoglobin concentration will be measured to the nearest 0.1 g/L, using a HemoCue Hb 301 system (HemoCue AB; Ängelholm, Sweden). The Eurotrol Hb 301 Control will be used to verify the precision and accuracy of the measuring device.

#### Physical Activity and Physical Fitness

Using the same protocol as for *KaziKidz*, physical activity behavior will be assessed with accelerometry. Cardiorespiratory fitness will be assessed through the Cooper 12-min run-walk test. The test is a simple, self-paced, maximal running test that is used to determine an individual’s maximal oxygen uptake (VO_2 _ max). The aim of the test is to run or walk as far as possible within 12 min. VO_2 _ max is then calculated with the following formula:

*VO*_2_ max (ml x kg^-1^ x min^-1^) = (*d*_12_ + 504.9)/44.73

where *d*_12_ refers to the total distance covered in 12 min in meters [50]. Before the test starts, blood pressure and heart rate are measured and a 10-min warm-up period is offered. All participants will receive the same instructions, and no verbal encouragement is allowed throughout the test. After the test is completed, a 5-min cool-down period will be given. Although all possible measures will be taken to reduce risk, all maximum exercise tests involve some risk. The test will be supervised by trained health care professionals with the necessary knowledge to deal with any medical emergency that may arise. Furthermore, an automated external defibrillator will be available on site.

Upper body strength will be determined with the handgrip resistance test utilizing the same procedure as described in the *KaziKidz* protocol.

#### Psychosocial Health Questionnaires

To gather information about the demographic profile and SES, health behaviors, and psychosocial health indicators of each participant, the following assessments will be completed by each participant by means of a questionnaire survey:

1. Demographic data and SES determined through household income and assets (property and car ownership).
2. Cigarette smoking, alcohol use, and screen time per day.
3. Subjective perceived health measured with 2 items from the 12-item short form health survey, adapted from the SF-36 [51]. Participants will be asked to rate the following questions: *In general, would you say your health is?* and *How motivated are you to improve your lifestyle?*
4. Work-related stress will be assessed using the short version of the original Effort-Reward Imbalance questionnaire [52].
5. The Shirom-Melamed Burnout Measure, a validated and widely used tool, will be used to assess occupational burnout [53].
6. Diet and nutritional analysis with a 24-hour dietary recall.
7. The General Health Questionnaire will assess mental distress or minor psychiatric morbidities [54].
8. Subjective sleep complaints will be assessed utilizing the brief 7-item self-report Insomnia Severity Index [55].

#### Data Collection and Statistical Analysis

The following data will be collected: (1) quantitative data on blood pressure, glycated hemoglobin and blood lipids, anthropometry and levels of physical fitness, cognitive performance and psychosocial health, (2) SES and demographic data, and (3) qualitative data on the feasibility and acceptability of the intervention measures implemented through FGDs. Quantitative data will be entered twice and cross-checked using EpiData version 3.1 (EpiData Association; Odense, Denmark). Cleaned data will be transferred to STATA version 13.0 (STATA Corp, College Station, TX, United States of America). Questionnaire data will be collected using the software package EvaSys (Survey Automation Suite, version 7.1) and analyzed with STATA.

Clinical and anthropometric indicators, physical fitness, cognitive performance, and psychosocial health values will be summarized by their mean and SD at normal distribution and otherwise by their median and interquartile ranges. Questionnaire information on psychosocial health will be expressed as a percentage.

For the analysis of cross-sectional and longitudinal associations, mixed linear or mixed logistic regression models will be used, depending on the type of outcome variable. These models will be adjusted for clustering within classes and schools using random intercepts. In analyses of cross-sectional associations, the models will include personal characteristics of children, such as gender and age, SES of parents or guardians, and other potential confounders of the associations of interest. Models assessing intervention effects will additionally include 3 indicator variables, as defined at the level of schools, 1 for schools of the intervention arm, 1 for schools receiving teacher workshops, and 1 for schools receiving teacher coaching. In addition, these models may include the value of the respective outcome variable at baseline. As intervention effects may also depend on the child’s initial characteristics, stratified analyses and analyses with interaction terms will be performed. Potential effect modifiers to be tested include gender, age, SES, ethnicity, health status, and physical fitness at baseline.

The primary objectives of the statistical analyses are (1) to assess the physical fitness of the participants and their associations with cognitive performance and psychosocial health at the beginning and over the course of the intervention, and (2) the effect of interventions on disease status and other health parameters. The secondary objective is to assess the feasibility and acceptability of the health interventions, as determined by FGDs.

### Availability of Data and Materials

The datasets generated and/or analyzed during the present study are not publicly available due to confidentiality but are available from the corresponding author on reasonable request.

## Results

### Overview

The project was funded in April 2017 and enrollment of the participants was completed in January 2019. The baseline survey was conducted from January to March 2019. At the time the present paper is being written, the *KaziKidz* and *KaziHealth* intervention are underway. The follow-up survey is planned for September to October 2019. At the end of the study, the results will be communicated to the Department of Health and the Department of Education in Port Elizabeth, as well as the involved schools. All intervention materials will be made available to the control schools after completion of the study. Workshops will be offered to the control schools to prepare teachers to implement the *KaziKidz* teaching material. Furthermore, teachers of the control schools will have the possibility to take part in the workplace health promotion intervention program after the completion of the second health assessment. The key findings will be submitted for publication to the peer-reviewed literature and presented at national and international conferences.

## Discussion

### Principal Findings

Results from the DASH study revealed that the prevalence of soil-transmitted helminth infections among grade 4 children was above 60% (90/149) in several schools in Port Elizabeth [56]. Moreover, infected children had lower VO_2_ max compared with their noninfected peers [56]; helminth infections and low physical fitness were significant predictors of low selective attention and poor academic achievement [16]; physical activity was associated with health-related quality of life [57]; almost one-third of all school children were classified as hypertensive [58]; and the physical activity intervention component contributed to the maintenance of academic performance [27] and resulted in a significantly delayed increase in children’s BMI [59]. Importantly, the DASH intervention package was well received in all schools.

The *KaziBantu* project is a logical continuation and expansion of the DASH project and aims at contributing to healthy schools and healthy communities. Teachers, as leaders in communities, have an important role to play in this regard. We conjecture that teachers as healthy role models will be able to promote better health behaviors and encourage a healthy, active, and inspiring environment for learners and peers at school. Various health professionals will empower teachers with specific knowledge related to infectious and NCD risk factors, physical activity and fitness, and psychosocial health and nutrition. Improved health and well-being increase teachers’ productivity, benefiting their own health and well-being and that of the children they teach and educate. We hypothesize that implementing *KaziBantu* will result in less absenteeism, a reduction in stress, and better coping with work demands.

Pursuing the present study protocol will provide specific answers to the following questions: Are *KaziKidz* teaching materials useful? What are the difficulties in using the teaching materials from the perspective of the teachers? What are the teachers' experiences with regard to the coaching by the teacher coaches? What experiences do the coaches have in their work with the teachers? What attitudes do teachers have with regard to the lessons proposed? What are the conditions for an effective and sustainable implementation of this teaching material? Does the acceptance of the *KaziKidz* teaching material by the teachers moderate its effectiveness?

With regard to the implementation of *KaziKidz* and *KaziHealth*, 3 languages are spoken by the communities in the study area, namely, Afrikaans, IsiXhosa, and English. To ensure comprehension, questionnaires translated into the local languages have been pretested by native speakers, with an emphasis on those that focus on mental health indicators to match the educational attainment of children and help them to understand and answer the questions. The study will be conducted in impoverished and harsh environments where illiteracy, neglect, and violence are common [60,61], which might have an impact on the granting of informed consent by parents and guardians. For illiterate parents or guardians, a literate witness will be invited to sign, whereas participants will be asked to provide a thumbprint. To ensure return of the signed consent forms, we might ask potential study participants several times. Specific safety measures are in place to implement the research. Although it is difficult to predict the extent of people’s mobility and movement, we anticipate a substantial loss to follow-up as people show considerable mobility in this setting. Multiple imputations will be used to deal with missing data, as appropriate.

### Conclusions

Taken together, the *KaziBantu* project presented here builds upon the previous DASH study and aims to improve physical health and well-being, cognitive performance, and psychosocial and clinical health of children and teachers. The South African Department of Education seeks to create a lifelong learner who is confident, independent, literate, numerate, multiskilled, compassionate, and has respect for the environment and the ability to participate in society as an active citizen. The Department of Education also envisions healthy teachers who are qualified, competent, dedicated, and caring and who will be able to fulfill the various roles of an educator. Hence, the project aspires to assist the Department of Education by contributing to the development of the full potential of each learner and the transformation of education in South Africa. In addition, developed and validated *KaziKidz* workshop material may be translated into short learning programs for accreditation of Teachers’ Continued Professional Development. The *KaziChat* app will be made available to the Department of Education's directorate responsible for human resources for distribution to all teachers together with encouragement for implementation. This study builds on local evidence and offers the opportunity of providing new evidence on health intervention responses to NCD risk factors as a benchmark for future controlled studies that will enable comparisons among marginalized communities between South Africa and other African countries.

## Abbreviations

BMI: body mass index
CDC: Centers for Disease Control and Prevention
DASH: Disease, Activity and Schoolchildren’s Health
DXA: dual-energy x-ray absorptiometry
EKNZ: Ethics Committee Northwest and Central Switzerland
FGD: focus group discussion
HDL-C: high-density lipoprotein cholesterol
LDL-C: low-density lipoprotein cholesterol
LMIC: low- and middle-income country
MVPA: moderate-to-vigorous physical activity
NCD: noncommunicable disease
non-HDL: nonhigh density lipoprotein cholesterol
NTD: neglected tropical disease
PE: physical education
POC: point-of-care
RCT: randomized controlled trial
SES: socioeconomic status
VO_2_max: maximal oxygen uptake
WHO: World Health Organization
